# Development of semantic verbal fluency in children aged 2 to 5 and its relationship with participating in music activities

**DOI:** 10.1371/journal.pone.0350326

**Published:** 2026-06-24

**Authors:** Ritva Torppa, Valerie Looi, Pentti Henttonen, Seija Pekkala

**Affiliations:** 1 Department of Speech-Language Pathology, Faculty of Medicine, University of Helsinki, Helsinki‌‌, Finland; 2 Cognitive Brain Research Unit (CBRU), Centre of Excellence in Music, Mind, Body and Brain (CoE MMBB), Department of Psychology, Faculty of Medicine, University of Helsinki, Helsinki, Finland; 3 Spiral Therapeutics, San Francisco, California, United States of America; Bournemouth University, UNITED KINGDOM OF GREAT BRITAIN AND NORTHERN IRELAND

## Abstract

This cross-sectional study investigates the development of semantic verbal fluency performance (i.e., verbal recall) and the role of music participation in 79 normally-hearing Finnish children aged 2;0–5;11. The primary outcome measure was a semantic verbal fluency task where children listed as many animals or clothes as they could in 60 seconds. Additionally, parents filled in the “Role of Music in Families” questionnaire detailing their child’s formal and informal music activities. Results showed that the number of correct words belonging to the given category, along with the number of semantic clusters, subcategories and switches between subcategories, increased significantly with age. The rate of development between age groups differed for different aspects of verbal fluency, and more informal music activities was associated with better performance. These results indicate maturation of verbal recall from age 2;00 onwards, and that informal music participation is associated with the rate of development.

## Introduction

As part of our development of speech and language, we develop an internalized knowledge of the vocabulary of our language, called the mental lexicon (part of our semantic memory), where the knowledge of word meanings (e.g., “Paris is the capital of France”) and information or concepts associated with these words are stored [1,2]. When we want to refer to words related to specific object or category, we access our specific mental lexicon. According to Aitchison [3], this is a multi-faceted complex concept comprising the lemmas of words, the knowledge of the semantic-syntactic meaning of a word, and the knowledge of the phonological codes for the word (including phonemic and stress pattern) which culminates in the use of verbal recall to find the phonetic-articulatory gesture of the word. Verbal recall is inherent to communication, and enables us to generate narratives, express emotions/feelings, answer questions, and provide context to a communication event. Hence, the development of verbal recall is a critical part of the development of speech and language in children. The present study assesses the development of this skill in children aged 2 to <6 years using a semantic verbal fluency task, and the associations of music participation with this.

Semantic verbal fluency tasks have been widely used both clinically and for research to examine the ability to, and effectiveness of, access to the lexicon and verbal recall [4]. In a typical semantic verbal fluency task, one is asked to name as many words as possible belonging to a certain semantic category (e.g., animals, clothes) within a restricted period of time (e.g., 60 seconds) [4,5]. Performance on the task is related to vocabulary size and organization of the mental lexicon [6], processing speed [4], executive function (working memory, inhibitory control and flexibility) and the capability to articulate words clearly [7]. The semantic verbal fluency performance is operationalized by firstly counting the number of appropriate (correct) words generated to a given semantic category (quantity of performance) which gives information about the speed of lexical retrieval and hence the effectiveness of verbal recall, and secondly by analyzing the effectiveness of qualitative word search strategies used in the process of verbal recall [4,8]. Clustering is a qualitative strategy where related words are grouped together. A cluster can be formed by words that belong to the same semantic category (e.g., animals) or the words sharing a phonological similarity (e.g., words beginning with the same sound(s) ’dog’ and ‘dolphin’). In some studies, words that are both semantically and phonologically related (e.g., the Finnish words *kissa*, *koira*, *kani*, English translation ‘cat’, ‘dog’, ‘bunny’) have been separately counted as clusters [9,10]. The first outcome measure is the number of semantic, phonological and mixed clusters [6]. The second outcome measure is the mean cluster size. This is the mean of the number of the words in each cluster across all clusters [5,8,11–14]. In order to perform a semantic verbal fluency task, one needs to be able to both effectively search the lexicon for, and change the grouping strategy (e.g., from the subcategory of sea animals to the subcategory of farm animals, or grouping words that share the first phoneme, e.g., ‘cat’, ‘camel’). The outcome measure of this qualitative strategy called ‘switching’ is counting the number of switches between subcategories [4,8]. This reflects the executive flexibility of semantic verbal fluency performance. The third qualitative strategy is to produce words from a variety of subcategories. The effectiveness of this strategy is assessed by counting the number of different subcategories included in the clusters the child formed during the task, reflecting the scope and diversity of one’s semantic knowledge and the ability to combine this information stored in the semantic memory [4,15]. All of these strategies are thought to require higher level executive functions such as working memory, inhibitory control and cognitive flexibility [7,8,16].

Previous research has consistently demonstrated a link between children’s quantity of semantic fluency performance and various language skills, including receptive vocabulary [17,18], expressive vocabulary skills (the ability to name pictures and provide synonyms) [6], verbal comprehension, syntax comprehension, and sentence repetition [17]. Additionally, research has shown that performance on verbal intelligence assessments is associated with both the quantity [17,19,20] and quality of children’s semantic verbal fluency, such as the number of clusters, average cluster size, and the number of switches [20].

While the role of executive functioning skills in semantic verbal fluency performance is well-established in adults [4], research on young children is less conclusive. For children aged 4–5 years, Chami et al. [6] found that both the quantity and quality of semantic verbal fluency performance were primarily influenced by expressive vocabulary skills (as defined above), rather than by executive skills which were calculated in their study as a composite score for working memory, non-verbal switching, and inhibition. Ruffini et al. [7] reported that in children aged 3–6 years, the variance in the quantity of semantic verbal fluency performance was explained by composite and individual scores for visual working memory, inhibitory control, and flexibility – all executive functioning skills. Similarly, Bisevic et al. [21] found that for children aged 3:0–6:0, better performance in their executive functioning task, the Dimensional Card Sorting Test, was associated with improved quantity of semantic verbal fluency performance. It is not yet clear how and why semantic verbal fluency performance develops in children, and the exact trajectory of this development. Previous studies have shown that the number of correct words produced by children in a semantic verbal fluency task increases with age from 3 to 15 years [6,22–25], explained by a growth in vocabulary knowledge and progressively higher levels of cognitive functioning [5,6,22]. The number of clusters and switches has also been found to increase with age in children over 4 years and at school age [5,6,26–29]. The impact of maturation on mean cluster size is less conclusive [29]. For instance, Koren et al. [30] and Karousou et al. [31] found that in children aged 8–11 years and 4–16 years (respectively) did not improve in their mean cluster size as they got older. In contrast, Hurks et al. [14] found that mean cluster size increased in children from age 7 to age 8–9, as did Kavé et al. [13] whose results were for participants aged from 8 to 29 years. However, very little research has been conducted on the qualitative word search strategies used by children under school age, especially in the important peri-lingual stage from age of 2–4 years, and to our knowledge, there are no studies on children under the age of 3 (see for instance [29]). It is therefore unclear to what extent typically developed young children utilize qualitative word search strategies (clustering, switching or forming semantic subcategories) in semantic verbal fluency tasks and the degree to which the acquisition of successful verbal recall requires the use/development of word search strategies or executive functions in the early years. It is also not known if the children aged 2–3 years use clustering or switching, and/or form semantic subcategories, nor when these strategies emerge in childhood.

Whilst children’s verbal recall often allows fluent communication with others, this is not the case for many children. For instance, beyond the typically-developing child, another body of research has shown that children with hearing deficits struggle with verbal recall as shown with poor performance in word finding tests and in semantic verbal fluency tasks (e.g., [32–34]), similar to children with specific language impairments [19]. Thus, it is important to identify factors that may contribute to children’s verbal recall performance in order to find ways to help especially children struggling with it. For instance, it has been found that semantic verbal fluency performance is better in children whose parents have higher educational levels ([35,36], among others). However, socioeconomic status and parental education is not a variable that can be easily changed or used as a rehabilitation method. It would be important to find if some ‘common’ or easily accessed activities are linked to improved semantic verbal fluency performance as this may help access and participation in evidence-based appropriate intervention activities. One such activity could be music, given that music and language share sensory, cognitive, and neural resources and there are robust associations between music and language abilities starting from early childhood [37]. The OPERA (Overlap, Precision, Emotion, Repetition, Attention) hypothesis proposes that even non-linguistic musical activities can lead to adaptive plasticity in speech-processing networks as well as improve language skills [38] and therefore also semantic verbal fluency. The assumptions underlying this include that: 1) there is anatomical overlap in the brain networks that process acoustic features used in both music and speech, 2) music places higher demands (precision) on these shared networks than speech, 3) adaptive plasticity can happen if the musical activities that engage this network elicit strong positive emotion, (4) the musical activities are frequently repeated, and (5) the musical activities require focused attention [38].

Music participation can be both formal in nature (e.g., music lessons, singing in choirs, playing in bands/orchestras, undertaking pedagogic music programs (e.g., Kindermusik, Yamaha or dance lessons), or more informal in nature (e.g., listening to music, participating in social music activities with friends or family, watching and listening to music from videos, online games or television, independent music exploration, dancing/singing to music etc.) [39–42]. In line with the OPERA-hypothesis, previous studies in adults have provided some evidence on the effect of repeated, engaging and attentive music activities on semantic verbal fluency. Lyu et al. [43] found in a randomized controlled trial in adults with mild Alzheimer’s Disease that music therapy including singing and listening to familiar songs improved semantic verbal fluency, whilst this was not the case for the control group who read the lyrics of the songs. Further, Moisseinen et al. [44] found in 95 adults (aged 21–88 years) that the more the adults participated in choir singing, the better their semantic and phonemic verbal fluency.

Within the pediatric population, there is some evidence on improved semantic verbal fluency with formal music activities, largely attributed to children having to actively attend and engage in the activities the leader introduces for them. This is consistent the assumptions of OPERA-hypothesis. Janus et al. [45] pseudo-randomly (to assure that the average score on the background measures for each group were similar) assigned their cohort of 4–6 years old children to either receive French training (28 children) or computer-based music training (29 children), finding that both types of training resulted in better semantic verbal fluency after training when compared to pre-training. Studies also report better verbal fluency with more music training in children aged over 4 years. Zuk et al. [46] showed in a cross-sectional study better semantic and phonemic verbal fluency in 15 musically trained children when compared to 12 non-musically trained children (all aged 9–12 years). Existing research has also reported associations between formal music activity participation and better scores on tasks evaluating language skills related to verbal recall and semantic verbal fluency performance. Linnavalli et al. [47] studied 66 children who participated in music playschool (28 children), dance playschool (32 children; active control group) or regular day care activities (passive control group), and followed them for 2 years. They found that children in the music playschool improved significantly more than other groups in verbal intelligence measured with a WISC vocabulary task, similar to the task in Mengisidou et al. [17] and Henry et al. [19] (see above). Jaschke et al. [48] randomly assigned 147 children aged 6 years to receive either music or visual art training. They found that verbal intelligence measured with the same WISC vocabulary task as Linnavalli et al. [47] improved significantly only in children participating in music training. This replicated the results of Moreno et al. [49] who assigned (not randomly) 64 children, aged 4–6 years, to interactive computer-based music training or visual art training groups and found that only children in the music group improved significantly in the measure of verbal intelligence. A meta-analysis on music training studies involving children aged 3–11 years that included a control group also reported that the research evidence supports that music training improved executive functioning, specifically inhibition control related to semantic verbal fluency performance [50]. The effect size was moderate-to-large for the 8 randomized controlled trials and small-to-moderate for the 22 longitudinal studies altogether included in the meta-analysis [50].

As said above, the OPERA-hypothesis proposes that if musical activities are frequently repeated and engaging, they can lead to improvement of speech processing and further, language skills. Thus, since children participate in informal music activities more regularly than formal activities and enjoy them [41,42], it may be that improvements in semantic verbal fluency performance could be driven more by informal, than formal music activity participation. Should this be the case, benefits of this include that informal music making/singing is far easier to participate in, much more accessible for most parents/families, and usually cheaper as well. Prior to this study, the possible impact of informal music participation has often been overlooked with studies primarily accounting for only music training or formal music activity involvement. According to our best knowledge, there are no studies on children assessing directly the links of informal music activities to semantic verbal fluency performance. However, Torppa et al. [51] found that in 18 children with hearing loss, aged 3–6 years, singing pitch accuracy improved with better quantity of semantic verbal fluency performance and more informal music activities. Since the connections were probably bi-directional, the authors concluded that good singing skills along with informal music activities, could support semantic verbal fluency. For skills associated with semantic verbal fluency performance (see above), Torppa et al. [34] found that participation in formal and informal music activities with an emphasis on singing was a significant predictor of verbal intelligence for 5−13 years old children with normal hearing (NH; *n* = 31) and with cochlear implants (CIs; *n* = 21). Papadimitriou and colleagues [52] found that more informal music activities predicted better receptive vocabulary in 64 infants younger than 18 months, and Schaal et al. [53] found that greater levels of music engagement was significantly associated with better expressive vocabulary in 118 children aged 20–26 months (although not significant in 86 children aged 32–36 months). Williams et al.’s [54] regression analyses of 3031 participants showed that participating in more informal music activities (singing, dancing, playing music instruments, etc.) shared with mothers or other adults at the age of 2−3 years was significantly associated with better receptive vocabulary when assessed later at the age of 4−5 years. Franco et al. [55] found that more informal parental singing with 26 6-month-old infants predicted better verbal comprehension outcomes in the second year, whilst Mustonen et al. [56] found in a cross-sectional study that greater levels of parental singing significantly predicted better expressive lexical skills and general expressive language ability for 164 children aged 2;5−4;1. Similarly, Torppa et al. [34] found that parental singing was also a significant predictor of verbal intelligence and word finding especially in children with CIs aged 5–13. One important factor related to the performance of any tasks is the ability to focus attention for a prolonged period [57]. Putkinen et al. [58] found that informal musical activity was associated with attention-related brain responses (P3as). Their results suggested that more informal musical activity participation was associated with a more mature auditory attention and better ability to suppress distracting sounds. Furthermore, in children with CIs aged 4–13, greater levels of informal singing at home was associated with improved development of attention-related P3a responses for changes in music and speech [59,60]; the development of these responses was poorer in children who did not sing or whose parents did not sing for them at home. These results further suggest that informal music participation and informal singing can potentially improve attention-related brain networks in young children. Overall, these studies provide preliminary evidence of potential relationships between informal music participation and semantic verbal fluency task performance in young children that warrant further investigation.

As alluded above, the intensity and perhaps also the nature of the informal music activities, especially with parental singing, might lead to stronger connections of informal than formal music activity to semantic verbal fluency performance in young children. Therefore, one limitation of current research investigating the connections of music participation to speech/language development is that they do not differentiate between formal vs. informal music activities, and the associations of these individually with a child’s semantic verbal fluency performance. Further, there is a paucity of research looking at the development of both the quantitative and qualitative verbal fluency performance in children in the critical peri-lingual age rate of 2;0–5;11 years prior to starting school, and according to our knowledge, there is no research on children aged 2;0–2;11. Hence the aim of this study is to investigate the development of both these aspects of semantic verbal fluency performance across the age range from 2 to <6 years, as well as the role of music participation in this development. The following hypotheses are proposed: Hypothesis 1: Children’s quantitative and qualitative semantic verbal fluency performance will improve with age. This will be measured by the following end points: a) the number of generated, correct words belonging to a given category (quantitative performance) and, b) the aggregated number of semantic, phonological and mixed clusters, the mean cluster size, the number of switches, and the number of semantic subcategories (qualitative performance). Hypothesis 2: Participation in formal and informal music activities will be associated with quantitative and qualitative semantic verbal fluency performance. Hypothesis 3: The associations between music activities and semantic verbal fluency performance will be stronger for informal than formal music activities.

## Materials and methods

This study was approved by The University of Helsinki Ethical review board in humanities and social and behavioral sciences (the number of approval: 43/2017) and all procedures were in accordance with this approval. Written informed consent was obtained from the parents of all participants, and recruitment occurred between 12.1.2018 and 31.3.2018.

### Questionnaire and procedure‌‌

The data was collected as part of an international research collaboration titled “The role of music in families of children with hearing loss and normal hearing in Australia, Finland, and the UK” [41]. As part of this, the anonymous electronic survey “the Role of Music in Families Questionnaire” (RMFQ) was distributed in Finland to the normal-hearing and hearing-impaired participants using Facebook advertisements. As only five children with hearing impairment performed the semantic verbal fluency task (which was only included in the Finnish version of the RMFQ), this cohort was excluded from the present paper as it was insufficient for statistical analyses. The RMFQ also collected background information, i.e., age, gender, maternal education level, paternal education level (Likert scale 1–6), family income (Likert scale 1–5), and information on the musical habits (i.e., parental and child’s own singing, informal and formal music activities) of children and their families (see [41]). See S1 File for the questions, response options and scales used in the present study), completed online using QualtricsTM.

### Participants

The participants were 79 normally hearing (NH), Finnish-speaking children living in Finland, aged 2;0–5;9 (35 girls, 44 boys; See Table 1) who had performed semantic verbal fluency task where they listed words for animals and clothes categories. The entire sample in Finland was N = 242 but only 79 of these children completed the verbal fluency task. All children had to have parent-report normal hearing, with no diagnoses, impairments or illnesses which could affect their speech, language or communication or musical skills (e.g., autism spectrum disorder, intellectual impairments, speech/communication disorders, blindness, certain syndromes etc.), as reported by the parents. Children were not to be attending elementary school (i.e., prior to entry into school), and had to have completed the verbal fluency task.

**Table 1:** Age range, gender and parental education of the participants in each age group.

Age group	Age range	*N* male + female (*n*F, *n*M)^a^	Education, mother^b^ *M* (*SD*)	Education, father^b^ *M* (*SD*)
2yr olds (2;0–2;11)	2;00-2;10	19 (10F, 9M)	4 (1.2)	3 (1.3)
3yr olds (3;0–3;11)	3;00-3;11	20 (10F,10M)	4 (1.2)	4 (1.4)
4yr olds (4;0–4;11)	4;00-4;10	21 (7F, 14M)	4 (1.4)	3 (1.3)
5yr olds (5;0–5;11)	5;00-5;11	19 (8F, 11M)	4 (1.3)	4 (1.4)

*N* = 79; ^a^*N male + female* = number of participants in each age group; *n*F = number of females; *n*M = number of males. ^b^Scale for education of mothers and fathers: 1 = primary school, 2 = middle school, 3 = lower university, 4 = middle university, 5 = upper university, 6 = graduate university (PhD) degree.

### Semantic verbal fluency task

The semantic verbal fluency task instructions (see S2 File) given to a child were based on the work of Troyer et al. [8] and Pekkala [9] and further modified by the first author of the present study. The parents recorded the child’s semantic verbal fluency performance and then transcribed the words their child produced over a 60-second time period, recording all of this information into the survey. Parents were clearly instructed how to perform the task, stipulating that they were to ask their child to list the names of animals and clothes (as 2 separate lists) from memory for 60 seconds. Parents were instructed not to prompt the child or show them any visual cues, but to encourage them to continue if they paused during the 60 seconds (see S2 File).

#### The coding and analysis of semantic verbal fluency performance‌‌.

To ensure coding was consistent across all of the children, all of the coding was done by a Masters research student who received training and written instructions for conducting the analysis from the last author of the manuscript, an expert in the field of verbal fluency. The coder was not blind to the condition “age group” as this information was required for her thesis, but the coder was blinded to the conditions “formal and informal musical activities”.

For both semantic verbal fluency categories, the coder was instructed to count the number of all of the words the child produced, and subsequently to aggregate the number of correct words for each given semantic category, to assess the quantitative semantic verbal fluency performance.

To further assess the qualitative semantic verbal fluency performance, the coder assessed the number of semantic, phonological and mixed clusters (clustering), the cluster size, the number of switches from a subcategory to another (switching), and the number of semantic subcategories used, pre-determined by the previous literature [8,9]. The semantic subcategories were determined based on the data, following the examples set in earlier studies [8,9]. At first, all of the words produced by the children, including those which did not belong to the given semantic category, were classified as belonging to either ‘clusters’ or to ‘individual words not forming a cluster’. The clusters were classified as ‘semantic’, ‘phonological’ or ‘mixed’ clusters [8–10]. As per the instructions for the coder, a semantic cluster contained at least two consecutive words belonging to the same semantic subcategory (e.g., ‘cat’, ‘dog’ in the subcategory of pets, ‘mittens’, ‘hat’, in the subcategory outerwear) [8]. A phonological cluster contained at least two consecutive words that started with the same phoneme, e.g., *aasi*, *ankka* (English translation: ‘duck’, ‘donkey’) or *hattu*, *hame* (English translation: ‘hat’, ‘skirt’) [8]. A mixed cluster was formed by at least two consecutive words sharing both semantic and phonological similarity (e.g., the Finnish words *kissa*, *koira*, *kani* (English translation: ‘cat’, ‘dog’, ‘bunny’) [9,10]. Words repeated in succession (e.g., ‘pants’, ‘pants’, ‘pants’) were not counted as a cluster [9].

According to the method of Troyer’s working group for the category of animals [8], and the method of Pekkala for the category of clothes [9], if a cluster simultaneously contained a larger and a smaller cluster, the coder was instructed to count only the larger cluster. For example, in the following list of words, ‘fish’, ‘gerbil’, ‘hamster’, ‘dog’, the words ‘gerbil’ and ‘hamster’ form a smaller cluster of rodents embedded in the bigger subcategory of pets and thus was instructed to be counted as its own cluster. If a word was linked to two clusters, the coder counted them as belonging to both clusters. For example, if a child listed words *kameli*, *koira*, *pupu*, *kilppari* (English translation: ‘camel’, ‘dog’, ‘bunny’, ‘turtle’), the word *koira* (English translation: ‘dog’) formed a phonological cluster with *kameli* (English translation: ‘camel’), and a semantic cluster (pets) with the words ‘bunny’ and ’turtle’ [8]. Semantic subcategories were defined based on the semantic relatedness of the words produced by the subjects, e.g., pets, farm animals, water animals, birds, insects, rodents, etc. [8] or coats, footwear, headwear, indoor/outdoor clothes, etc. [9].

As provided in the instructions for the coder, for the animal and clothing semantic verbal fluency categories, the number of clusters [8], mean cluster size, the number of switches [8], as well as the number of semantic subcategories [9] were calculated. The number of clusters contained the semantic, phonological [8] and mixed [9,10] clusters. For the count of cluster size, we started the count with the second word in a cluster, (i.e., a single word was given a cluster size of 0, two words had a cluster size of 1, three words had a cluster size of 2, etc.) as per the protocol introduced by Troyer [11]. Errors and repetitions were included. The mean cluster size was calculated by summing the size of each cluster and dividing by the number of clusters [11] and calculated separately for the animal and clothing categories. The number of switches was calculated as the number of transitions between clusters, including individual (single) words and errors [8]. The number of semantic subcategories consisted of the number of different semantic subcategories from which the children listed words for the category of animals or clothes [9].

To ensure the reliability of the scoring, a second assessor independently analysed 20% of the data produced for each category, after which the analysis results were compared with the results of the actual coder’s analysis of the data. The PPA method (point by point agreement) was used to calculate the assessor reliability accordingly: PPA = agreements for the trial/agreements for the trial + disagreements for the trial * 100 [61]. The percentage-agreement between the second assessor and the coder was 92% for the animal category and 82% for the clothing category. Subsequent to this, the second assessor and coder examined each of the discrepant evaluations discussing the scoring criteria (discussion-to-consensus), after which the final decisions for approval were made and the final scoring criteria adapted to all of the data as per the recommendations of Kazdin [61].

### Music activities

The present study used the following RMFQ questions assessing the frequency of participation in formal and informal music activities (see S1 File):

- Formal music activities: Music Lessons; Singing Groups; Instrumental Groups; Special children’s music programs; Dance classes; Other organized music programs or activities.
- Informal music activities: Listening to music informally (audio only); Social music activities; Musical videos; Family music activities; Music online (games, listening, etc.); Independent music exploration; Creating/making up songs or music performances for play or fun; Dancing informally; Live music concerts.
- In addition, the following questions were included in this study to evaluate parental singing: How often parents sang face to face with their child during the previous year, and during the child’s first year of life. We also asked in question “how often the child sang at home in general”.

An 8-point Likert scale was utilised for all music activity questions (0 = not at all; 7 = daily; see S1 File).

The responses to questions for formal music activities were summed together for each child (the sum variable herein called “formal music”; across all participants, *M* = 9.00, *SD* = 5.68, see S1 Table). Moreover, the responses for informal music activities were summed together. The initial intention was to examine the specific role of parental singing and singing by the child independently, but these variables were not normally distributed, with the following significant co-correlations found:

- “Parental singing face to face with the child during the previous year” and “parental singing face to face with the child during the first living year of the child” (*rho* = .030, *p* = .007);
- “Singing by the child during the previous year” and “parental singing face to face with the child during the previous year” (*rho* = .373, *p* ≤ .001), and
- “Parental singing face to face with the child during the previous year” and “singing by the child during the previous year” both correlated with the sum of the questions on informal music activities (*p* ≤ .010).

Therefore, the sum of responses to the questions on ‘informal music activities’, ‘parental singing with the child’, and ’singing by the child during the previous year’ was used to calculate the “informal music” score (across all participants, *M* = 52.72, *SD* = 7.71, see S2 Table), and used in analysis for hypotheses 2 and 3.

### Statistical analysis

The data were statistically analysed with SPSS 28 and R 4.5.2 software using a two-tailed significance value of 0.05.

To test hypothesis 1, the dependent variables of a) quantitative semantic fluency performance (the number of correct words) and b) qualitative semantic verbal fluency performance (the number of clusters, mean cluster size, the number of switches or semantic subcategories) were regressed on the age in years (continuous variable) using separate regression models for each dependent variable. To supplement these linear regressions, we compared age groups in order to determine if there were any differences between the age groups for semantic verbal fluency performance and the emergence of related qualitative verbal fluency strategies. The following age groups were used: 2;0–2;11 (“2yr olds”), 3;0–3;11 (“3yr olds”), 4;0–4;11 (“4yr olds”), and 5;0–5;11 (“5yr olds”).

As the Kolmogorov-Smirnov test showed that some of the verbal fluency variables were not normally distributed in the age groups, non-parametric testing was adopted for hypothesis 1 with differences between age groups examined with Kruskal-Wallis’ one-way analysis of variance followed by Dunn’s post-hoc test (with Bonferroni-corrections) where applicable.

Hypotheses 2 and 3 were tested for all participating children and for quantitative and qualitative verbal fluency variables using General Linear Modelling (GLM). These semantic verbal fluency variables were formed for statistical analysis: 1) number of correct words for animals and clothes summed together, herein called “quantity total”, and the following qualitative variables (which for each, were the sum of the items in the animal and clothing categories): 2) number of all semantic, phonological and mixed clusters “clusters total”; 3) number of switches “switches total”, and 4) number of all semantic subcategories “subcategories total”.

Before the actual analysis, maternal and paternal education (see S1 File for the questions, response options and scales for both) were summed together leading to sum score “parental education” (Likert scale, range 3.00–11.00, *M* = 7.25, *SD* = 2.21) which was added to the GLM model testing hypotheses 2 and 3 (see below) to avoid too many factors in the model. As six families did not answer the family income question, there was insufficient data on this variable to allow for inclusion in the model or sum score. All sum variables listed above and in the sub-section “Music activities” were normally distributed.

Notably, before actual GLM analysis we also inspected the correlations between age, “parental education”, “informal music” and “formal music” scores (see S3 Table). Age (continuous variable, range 2.00–5.91 years, *M* = 3.92, *SD* = 1.20) did not correlate significantly with music activity scores or parental education. Additionally, parental education also did not correlate significantly with any other independent variables included in the model. Informal music score correlated significantly with formal music score (*rho* = .403, *p* < .001), however this correlation was less than  .800 indicating that informal and formal music scores could be included in the same GLM model since the correlation lied within limits of acceptable multicollinearity [62].

In sum, the final GLM model to test hypotheses 2 and 3 included these dependent variables: 1) “quantity total”, 2) “clusters total”, 3) “switches total” and 4) “subcategories total”. The independent variables were: 1) age of the child (a continuous variable), 2) “parental education” (the sum of maternal and paternal education), (3) “formal music” (formal music participation score), and 4) “informal music” (informal music participation score). The first two of these factors were controlled in the GLM since they were expected to affect verbal fluency performance as discussed in the Introduction.

## Results

### Description of the data

The examples of children’s productions can be found in the S4 file. In all age groups, including the 2yr olds, there were children who could list animals and clothing items. Only seven parents reported that their child, who did not list any animals or clothes, did not understand the task, and all of these children were younger than 2;4 years.

As Table 2 shows, in both categories, the percentage of the correct words from the total number of words produced by the children increased considerably from the age group of 2- to 3yr olds, and for the animal category this increase continued to the age group of 5yr olds tested. For the clothing category, the percentage increased until the age group of 4yr olds and remained similar in 5yr olds (Table 2).

**Table 2:** The percentages (%) of correct words as calculated from total amount of produced words, and the means, ranges and standard deviations of correct words, semantic, phonological and mixed clusters, all clusters, mean cluster sizes, switches and semantic subcategories for each child in each age group and across all age groups (all children).

	% of correct words	N correct words *M (sd)*	N semantic clusters *M (sd)*	N phonol. clusters^a^ *M (sd)*	N mixed clusters *M(sd)*	N all clusters *M (sd)*	Mean cluster size *M (sd)*	N switches *M (sd)*	N subcateg.^b^ *M (sd)*
Animals									
2yr olds (*n* = 19)	51%	2.00 (1.94)	0.63 (0.68)	0.05 (0.23)	0.11 (0.32)	0.79 (0.92)	1.03 (1.35)	1.11 (2.05)	0.63 (0.76)
3yr olds (*n* = 20)	79%	5.75 (3.42)	1.00 (0.73)	0.30 (0.47)	0.10 (0.31)	1.40 (0.88)	0.90 (0.70)	3.40 (2.82	1.10 (0.72)
4yr olds (n = 21)	84%	9.38 (4.50)	2.24 (1.67)	0.38 (0.59)	0.14 (0.36)	2.86 (1.82)	1.27 (0.95)	4.76 (3.10)	1.91 (1.48)
5yr olds (*n* = 19)	93%	11.42 (4.75)	2.58 (1.58)	0.42 (0.61)	0.42 (0.69)	3.42 (1.74)	1.67 (1.28)	4.59 (2.78)	2.58 (1.30)
All children (*N* = 79)	82%	7.17 (5.18)	1.62 (1.48)	0.29 (0.51)	0.19 (0.46)	2.13 (1.75)	1.23 (1.08)	3.49 (3.04)	1.56 (1.33)
									
Clothes									
2yr olds (*n* = 17)	42%	1.00 (1.80)	0.24 (0.56)	0.00 (0.00)	0.11 (0.32)	0.29 (0.69)	1.21 (1.88)	1.78 (0.53)	0.24 (0.56)
3yr olds (*n* = 19)	64%	3.63 (2.24)	1.16 (0.77)	0.11 (0.46)	0.10 (0.31)	1.26 (0.99)	1.56 (1.60)	1.79 (1.69)	1.05 (0.62)
4yr olds *(n* = 19)	89%	8.95 (5.29)	2.58 (1.61)	0.11 (0.32)	0.14 (0.36)	2.90 (1.70)	2.04 (1.64)	2.79 (1.78	2.53 (1.58)
5yr olds (*n* = 19)	89%	8.94 (4.94)	2.28 (1.32)	0.22 (0.43)	0.42 (0.69)	2.56 (1.38)	1.93 (1.18)	2.83 (2.18)	2.17 (1.20)
All children (*N* = 73)	81%	5.71 (5.15)	1.59 (1.46)	0.11 (0.36)	0.19 (0.46)	1.78 (1.61)	1.77 (1.53)	1.93 (1.95)	1.52 (1.40)

^a^Phonol. = phonological. ^b^Subcateg. = semantic subcategories.

### Results for hypothesis 1

#### Quantitative performance.

**Fig 1:**
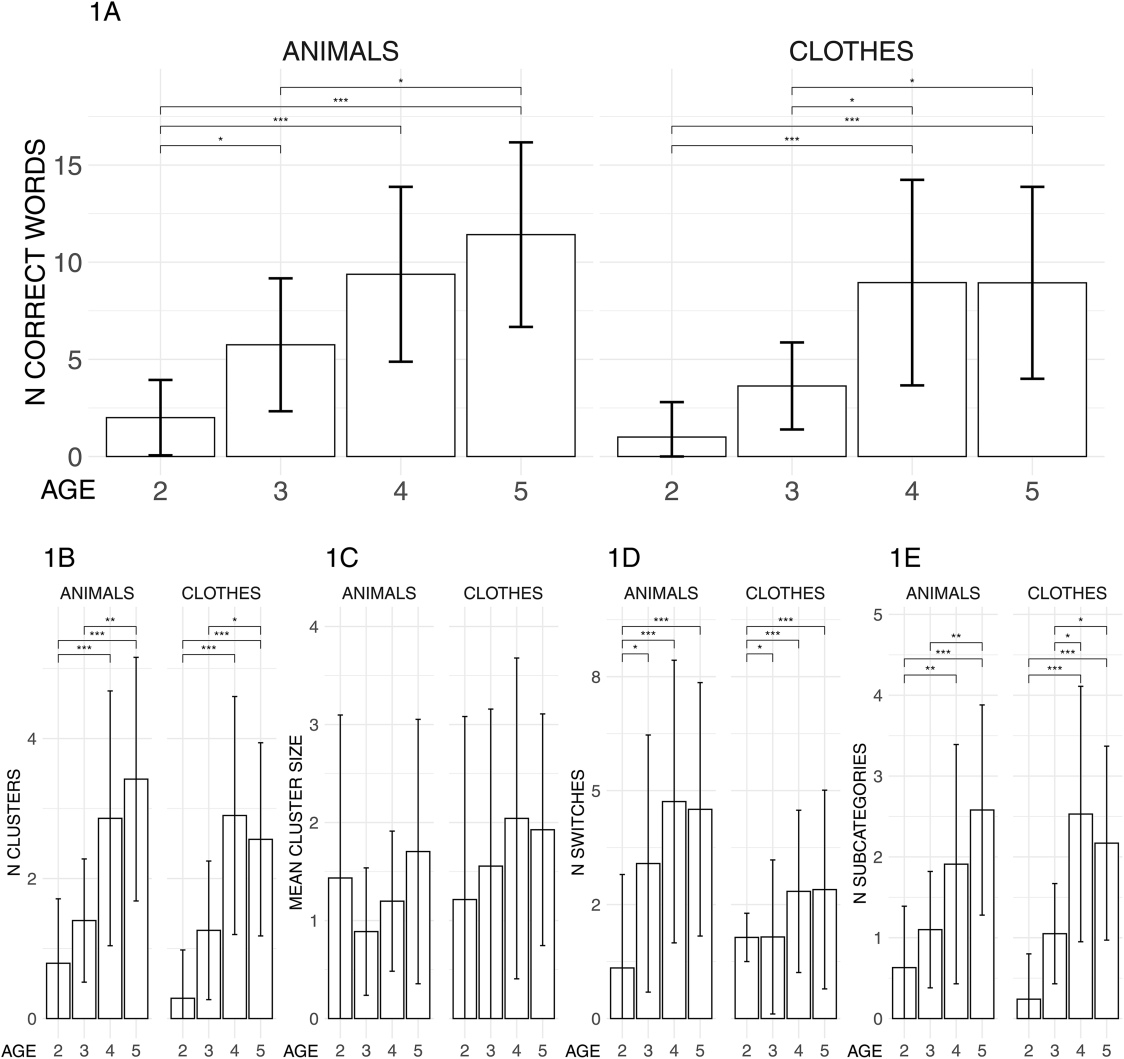
The Figs 1 A-E provide an illustration of the results for hypothesis 1, subdivided into the categories of animals and clothing, and given for quantitative (Fig 1A) and qualitative (Figs 1B-E) semantic verbal fluency performance for each age group (AGE, 2-, 3-, 4- and 5yr olds). Thus, the bar charts represent: Fig 1A) the mean of the number of correct words (N CORRECT WORDS) produced by each child in each age group; Fig 1B) the mean of the number of clusters (N CLUSTERS) produced by each child in each age group; Fig 1C) the mean cluster size (M cluster size) produced by each child in each age group; Fig 1D) the mean of the number of switches (N SWITCHES) produced by each child in each age group; and Fig 1E) the mean of the number of semantic subcategories (N SUBCATEGORIES) produced by each child in each age group. For each bar chart, the error bars represent the standard deviations of the children’s performance in each age group, and significant differences between age groups are highlighted with * as follows: * = p < .05; ** = p < .01; *** = p.001 (the significances are acquired from pairwise comparisons with post-hoc Dunn’s tests, adjusted with Bonferroni corrections, please see the corresponding supplementary S4-S7 Tables).

Overall, children produced on average 7.17 correct words for the animal category and 5.71 correct words for the clothing category (across all age groups) (Table 2). The regression analyses to test hypothesis 1 showed that the quantitative performance (number of correct words) increased with age (animals; *B(SE)* = 3.04(0.35), *p* < .001, *R*^*2*^ = 0.50; clothes; *B(SE)* = 2.64(0.39), *p* < .001, *R*^*2*^ = 0.39). The results for statistical analyses regarding age group differences are shown in Fig 1A and S4 Table. The Kruskal-Wallis test confirmed a significant difference in the number of correct words between age groups in both categories, for animals (*H* (3) = 41.405, *p* < .001) and clothes (*H* (3) = 35.614, *p* < .001). Post hoc -tests with Dunn’s test and Bonferroni-correction showed that 2yr olds produced fewer correct words than 4- and 5yr olds in both categories, and in the animal category, also fewer than 3yr olds (Fig 1A). 3yr olds produced fewer correct words than 5yr olds in both categories and 4yr olds in the clothing category. The difference was not significant between 4- and 5yr olds in either of the categories, between 3- and 4yr olds in the animal category and between 2 and 3yr olds in the clothing category (Fig 1A).

#### Qualitative performance.

The average (with standard deviations) number of clusters produced by the children in different age groups are shown in Table 2. The results for statistical analyses for age group differences are shown in Fig 1B and S5 Table. In both categories, the regression analyses showed that the number of clusters formed by children significantly increased with age (animals; *B(SE)* = 0.91(0.13), *p* < .001, *R*^*2*^ = 0.39; clothes; *B(SE)* = 0.74(0.13), *p* < .001, *R*^*2*^ = 0.31). Consistently, the number of clusters formed by children significantly differed between age groups (animals; *H* (3) = 30.433, *p* < .001; clothes; *H* (3) = 35.504, *p* < .001). Post hoc -tests with Dunn’s test however showed that in both categories, 2yr olds formed fewer clusters than 4- and 5yr olds, and 3yr olds formed fewer clusters than 5yr olds while no significant differences between 3- and 4yr olds or 4- and 5yr olds were found (Fig 1B).

The mean size of clusters (with standard deviations) produced by children in different age groups are also presented in Table 2. The results for statistical analyses for age group differences are shown in Fig 1C and S5 Table. Against our hypothesis 1, age did not predict average cluster size in animal (*B(SE)* = 0.18(0.11), *p* = .130, *R*^*2*^ = .033) or clothing categories (*B(SE)* = 0.25(0.19), *p* = .185, *R*^*2*^ = .030). Similarly, there were no significant differences between age groups (see S5 Table).

The number of switches produced by children in each age group is presented in Table 2. The results for differences between age groups are shown in Fig 1D and S6 Table. In both categories, the regression analyses showed that the number of switches significantly increased with age (animals: *B(SE)* = 1.18(0.26), *p* < .001, *R*^*2*^ = 0.22; clothes *B(SE)* = 0.78(0.17), *p* < .001, *R*^*2*^ = 0.23), and for both verbal fluency categories, there were significant differences between age groups (animals *H* (3) = 21.735, *p* < .001; clothes *H* (3) = 26.516, *p* < .001). Post hoc -tests with Dunn’s test showed that 2yr olds produced fewer switches than 3-, 4- and 5yr olds whilst the differences between other age groups were not significant (Fig 1D).

The average (with standard deviations) number of semantic subcategories produced by the children in different age groups are shown in Table 2. The most frequently used semantic subcategory of the animal category was exotic animals (e.g., ‘boa’, ‘lion’, ‘giraffe’, ‘elephant’), In the clothing category, the most frequently used semantic subcategory was indoor clothes (e.g., ‘shirt’, ‘pants’, ‘socks’, ‘leggings’). Both categories contained semantic subcategories that were used by only one or two participants. In both categories, the regression analyses showed that the number of semantic subcategories formed by children significantly increased with age (animals; *B(SE)* = 0.65(0.10), *p* < .001, *R*^*2*^ = 0.34; clothes; *B(SE)* = 0.63(0.11), *p* < .001, *R*^*2*^ = 0.30). The results for statistical analyses on the differences between age groups are shown in Fig 1E and S7 Table. The Kruskal-Wallis test confirmed a significant difference in the number of semantic subcategories between age groups (animals *H* (3) = 26.813, *p* < .001, clothes *H* (3) = 36.824, *p* < .001). Post-hoc tests with Dunn’s test showed that in both categories, 2yr olds formed fewer semantic subcategories than 4- and 5yr olds, and for clothing category, 3yr olds formed fewer semantic subcategories than 5yr olds with no significant differences between 4- and 5yr olds found (Fig 1E). Moreover, in the clothing category, 3yr olds formed fewer subcategories than 4yr olds (Fig 1E).

### Results for hypotheses 2 and 3

As the S2 Table shows, children often participated in informal music activities, the most common of these being parental singing, singing by the child and listening to music informally (on average > 4–6 times a week) (see S1 File for the questions, response options and scales for music activities). In contrast, participation in formal music activities was more infrequent averaging less than once a month, with the exception of singing in groups (mean participation: 2–3 times per month) (see S1 Table).

The GLM analysis showed that, apart from controlled factor of age (Wilk’s λ, *F*(4,60) = 15.32, *p* < 0.001), only the informal music score was significantly linked to verbal fluency quantity total (the sum of the number of words generated in both categories, *F*[1,63] = 5.51, *p* = .022), and also to subcategories total (the sum of subcategories in both categories, *F*[1,63] = 4.25, *p* = .043), although the multivariate test was not significant (Wilk’s λ, *F*(4,60) = 1.41). Semantic verbal fluency performance improved with more informal music activities. A trend between the informal music score and clusters was observed, but not significant (*F*[1,63] = 3.61, *p* = .062). The connection of formal music score to semantic verbal fluency variables was not significant (p ≤ .205).

There was a significant connection of age to quantity total and quality variables (p < .001; the connections of “parental education” to these was not significant, p ≥ .712). Moreover, as the S1 Table shows, children in the older age groups tended to participate more in formal music activities than children in younger age groups, and a trend between the continuous variable age and formal music score was observed, although not significant (*p* = . 066; see S3 Table). To test if age moderated the results on music activities, individual post-hoc GLM models with the three significant (*p* < .10) dependent variables were investigated with the inclusion of the interaction terms between age, informal and formal music. None of the interactions were significant, indicating that the effects do not differ according to participant age. Moreover, the‌‌ mediation of the strongest effect (informal music to quantity total) by age was tested with a formal mediation analysis (average causal mediation analysis [63]). There the effect due to age was not significant (*R*^*2*^ = .033, *p* = .748), accounting for only 9 per cent of the total effect (*R*^*2*^ = .356). Likewise, the modulation was not significant in case of formal music (*R*^*2*^ = .202, *p* = .228). This suggests that age did not mediate the results on music activities.

## Discussion

The present study investigated if the quantitative (number of generated correct words belonging to a given category) and qualitative (number of semantic, phonological and mixed clusters altogether, mean cluster size, number of switches, or number of semantic subcategories) aspects of semantic verbal fluency performance improved with age and/or with more formal or informal music activities in children aged from 2;0–5;11yrs. In line with our first hypotheses, the regression analysis showed improvement as children got older, and the age-group comparisons indicated overall differences between our cohorts of 2-, 3-, 4- and 5yr old children in both quantitative and qualitative (the number of clusters, the number of switches, and the number of semantic subcategories) performance. There was no difference between the 4- and 5yr olds, and against our hypothesis 1, there was no age-related development for the mean cluster size. In line with our third hypothesis, only informal music activities were associated with better quantitative and qualitative semantic verbal fluency performance.

### Quantitative semantic verbal fluency performance improves with age

The regression analysis and age group comparisons showed that the number of correct words in both semantic verbal fluency categories increased significantly from age 2;0 through to 5;11, consistent with our first hypothesis. Previous research in children aged 3 years and older showed similar results [6,22,23,25], with the current study suggesting that this development is likely to start from at least age 2 years.

As Fig 1A shows, 2yr olds produced fewer correct words than 4- and 5yr olds in both semantic categories, and in the animal category, also fewer than 3yr olds. Moreover, 3yr olds produced fewer correct words than 5yr olds in both categories and 4yr olds in the clothing category. There was no difference between 4- and 5yr olds in either of the categories. This suggests that in the animal category, there is a rapid development at age 2–3, which continues through to age 5, but at a slower rate between age 4- and 5 yrs. Previous research suggests that lexical semantic links necessary for semantic verbal fluency performance emerge during the second year of life and become more robust at age of 2 years and beyond [64]. It is also known that quantitative performance is linked with various language skills (receptive vocabulary [17,18], vocabulary skills, e.g., the ability to name pictures and provide synonyms [6], verbal comprehension, syntax comprehension, and sentence repetition [17]) and verbal intelligence quotient [17], and that language skills develop considerably from age 2;0–5;11 [65]. Thus, it is evident that the development of children’s lexical-semantic links and language skills play a role in the development of quantitative semantic verbal fluency performance of the children participating the current study. Notably, all of the children aged 2;4 or older were able to perform the task. Future studies should confirm if this age is important for the ability to understand and perform the semantic verbal fluency task.

For the clothing category, although the regression analysis showed that the development was similar to the animal category, the results between age groups indicated that development either started later and/or was slower in the 2–3yr olds. Fig 1 also shows that children tended to list more animal words than clothing words, aligning with existing studies showing that living categories (such as animals or fruits) elicit more responses compared to manmade (non-living) categories such as clothes [4]. It should also be considered though that the animal category has a larger item pool (i.e., number of items that can be named) when compared to the clothing category [4]. The later developmental pattern for the clothes than animal category could be a consequence of the animal words being more familiar to young children due to a greater exposure in the early stage of life. For example, early parental interaction often involved shared picture book reading including animal pictures, or imitation of animal sounds or animal names. Exposure to words related to clothing may occur slightly later, and to a lesser extent meaning that children learn, and learn to produce, clothing words later in life than animal words. This would consequently result in later development in finding the semantic-syntactic meaning, phonological codes, and/or phonetic-articulatory gestures for these words – processes that a child needs to undertake in order to find the correct words for a naming-based semantic verbal fluency task [3].

Another consideration is that the three main areas of executive function – working memory, inhibitory control, and cognitive flexibility – have been shown to independently explain performance in verbal fluency tasks in 3–6yr old children [7]. Inhibition is the ability to suppress automatic responses or interfering stimuli coming readily to mind, such as words not belonging to a given semantic category [7]. It is one factor in the attentional control domain which includes the capacity to selectively attend to specific stimuli whilst simultaneously inhibiting prepotent responses, the ability to focus attention for a prolonged period, and attentional control (the regulation and monitoring of actions so that plans are executed) [66]. The prefrontal cortex involved in these executive functions is already operative at the age of one, and previous evidence suggests that attentional control domain develops earlier than the other executive functions with development starting early on and progressing rapidly until age 5 years [66,67]. Ruff and Capozzoli [57] reported that duration of focused attention was relatively low in young children (aged 10 months to 2;2 in their study), but substantially increasing from age 2;2–3;6. This is also consistent with our findings that show the development of the quantitative semantic verbal fluency performance appearing by at least age 2 and continuing thereafter.

### The improvement of qualitative semantic verbal fluency performance by age

Interestingly, we found for the first time that already 2yr olds could form clusters, switches and semantic subcategories. Thus, many children use the qualitative strategies we measured already at age of 2 years as discussed later in this section. In all age groups, most clusters were semantic, indicating that word searching is primarily based on semantic connections in the verbal fluency task used in this study, similar to Gonçalves et al.’s [68] and Tallberg et al.’s [5] findings in older children. The low number of phonological and mixed clusters could be at least partially explained by the underdevelopment of phonological awareness and letter knowledge in children under school age [5].

When looking at the qualitative strategies employed by children as related to hypothesis one, regression analysis showed increase of the number of clusters from age 2;0–5;11. Both categories resulted in a similar number of clusters and the same significant differences between age groups, as shown in the Fig 1B. Two yr olds formed significantly fewer clusters than 4- and 5yr olds, and 3yr olds formed significantly fewer clusters than 5yr olds, with no differences between 3- and 4yr olds or between 4- and 5yr olds. Thus, for the number of clusters, these results indicate that development of the number of clusters is likely to start around (if not before) the age of 2, continuing through to at least age 5 (if not beyond). In fact, existing research shows that this development continues past the age of 5 with [5,26–28], all finding that number of clusters produced increases in children beyond age 5. This current paper though is the first one to provide evidence to indicate that the development of clustering, measured as the number of clusters potentially occurs at a far younger age than shown thus far in existing research.

In contrast to the number of clusters, we did not find any age-related development for the mean cluster size. Former studies show conflicting results, with some finding age-related improvements while others do not [29]. Our results align with those of Kavé et al. [13] in children and adults aged 8–29, Koren et al. [30] in children aged 8–11, and Karousou et al. [31] in children aged 4:0–16:11, all of whom also failed to find any age-related development in mean cluster size. We suggest that our finding regarding the lack of development in 2- to 5yr olds, along with previous findings in older children, supports the notion that cluster size is primarily influenced by the automatic activation of the lexico-semantic network, rather than by more age-related, controlled executive search processes [13,29], or by vocabulary and language development. In contrast, the number of clusters may increase since it reflects strategic or controlled searching [29], or is affected by language development.

In the current study, some of the 2yr olds switched between semantic subcategories, even though the number of switches was small. As would be reasonable to expect, 2yr olds produced fewer switches between subcategories than 3-, 4- and 5yr olds for both animal and clothing categories, however, the differences between any other age groups were not significant. Previous research has shown that the number of switches increases with age in children aged 4 years and older. This has been found in children aged 6–15 years in semantic verbal fluency for animals [5], in children aged 4;0–5;11 years for animal and food categories [6], and in children aged 5–15 years for the animal category [27]. The current study has found that for some children, switching has already emerged by age 2, but is generally more prominent at older ages (3–5 years). Switching in adults is thought to reflect executive functions as it requires a person to search the lexicon for, and subsequently change, the grouping strategy (e.g., from sea animals to farm animals) applicable to the task [4,8,16]. From the three main areas of executive function, switching in adults’ semantic verbal fluency performance particularly reflects the functioning of cognitive flexibility [4,8], however there is no consensus in the age when this skill becomes operative. Some studies suggest that cognitive flexibility emerges only around the age of 6 [67], however, Ruffini et al. [7] found that switching performance correlated with the number of correct words in the semantic verbal fluency task in children aged 3–6 years. Emerging cognitive flexibility might explain the variability in our ‘switching’ results.

The differences between age groups were similar for the number of clusters and for the number of semantic subcategories expect that 3yr olds also formed significantly fewer subcategories than 4yr olds in the clothing task. Thus, also for the number of subcategories, these results indicate that development is likely to start around the age of 2, continuing through to at least age 5 (if not beyond). According to Troyer [8], clustering involves accessing and using the mental lexicon. Moreover, the semantic subcategories reflect the scope and diversity of the semantic knowledge corresponding to one’s representation of a category as well as the ability to flexibly combine the information stored in the semantic memory [4]. Therefore, the higher mean number of semantic clusters than phonological clusters in these children can be at least partially attributed to finding the semantic meanings of a words [3]. It has been consistently found that for 3 and 4 year old children [23], and for 4–5 year old children [6,23], vocabulary is a predictor of scores in semantic verbal fluency tasks. Moreover, at the age from 3 to 4 years, there is a significant improvement in children’s lexical skills and vocabulary [69], and the differentiation of representations of the animal concept in 4–7 year old children [70]. Our results showing age-related improvement from 3 to 5 years in the number of clusters and semantic subcategories may be at least be in part explained by the development of semantic knowledge and its organization in semantic memory across this age range. Furthermore, the role of executive functions, including working memory, should not be ignored. Working memory processes are needed for word searching and forming both clusters and semantic subcategories, and it is known that working memory develops from 3 to 5 years of age [67]. Thus, we cannot exclude the role of working memory in the present developmental results on qualitative aspects of semantic verbal fluency.

It was also found that the subcategory development is unique to the individual as every child had a different range of subcategories (i.e., no 2 children named the exact same subcategories), suggestive that upbringing/‘nurture’ (as opposed to just ‘nature’) plays a significant role in early subcategorical development.

### More informal music activities are associated with better semantic verbal fluency performance

In addition to older age, higher levels of informal music activity involvement was associated with better semantic verbal fluency quantity and quality (particularly the number of subcategories). Our findings are consistent with the OPERA-hypothesis [38], and add to the literature reporting an association between higher levels of music participation and better performance on semantic verbal fluency, performance in tasks assessing language [34] and verbal intelligence [47–48], and associations of more informal music activity participation with better receptive vocabulary [52], better expressive vocabulary [53] and improved attention-related brain responses [58–60]. More informal music activities shared with parents or adults in the family (e.g., playing music, singing songs, dancing, and other music activities) at age of 2–3 have also been correlated with better receptive vocabulary and attentional and emotional regulation in children aged 3–4 years [54].

We have shown that the relationship between semantic verbal fluency performance and music participation is stronger for informal music activities than for formal activities, which according to OPERA-hypothesis [38] can be related to the higher level of informal music activity compared to formal music activity. Our finding could also be consequence of the differences between the nature of the formal and informal music activities. Home music activities provide a structure within which parents and children engage in mutually responsive interactions requiring joint attention, active cooperation, turn-taking, and immediate feedback [54] which are important for language development. As explained in the Introduction, previous evidence suggests that informal singing could have a positive effect on language skills underlying verbal fluency improvement [55–56], and in children with hearing loss, improved singing of pitch patterns is linked to both more informal music activities and to better semantic verbal fluency [51]. The results of this study potentially provide further evidence on the role possible importance of singing alongside other informal music activities in early pediatric development. Informal music activities create opportunities for children to use, practice and refine their spoken language and social skills, and are common to everyday life [41,71]. For example, social and family activities often include music and/or singing, or dancing to music. Many children watch TV shows that involve music or music groups for children, and they will sing along and/or talk about these shows/songs/groups to others. Thus, even seemingly ‘passive’ music activities such as listening to music may result in active, attentive and intensive music participation that includes the use of language, with the child singing, humming or talking about the music/lyrics etc. [71]. It is also known that young children enjoy music activities. Thus, informal music activities in the present study fulfill the perquisites for the adaptive plasticity leading to better language skills. They elicit strong positive emotion, are frequently repeated and are associated with focused attention.

This paper is the first to show an association between informal music activities and the quality of verbal fluency performance, particularly the number of semantic subcategories the children produced. One possible reason for this relationship is that the informal music activity/ies has exposed the child to a larger and wider range of words/vocabulary, thereby increasing the scope, quantity and diversity of a child’s semantic knowledge related to a particular category [4]. As Williams et al. [54] and Barret [71] pointed out, songs targeted for young children include action songs including body parts (e.g., Head, Shoulders, Knees, and Toes), songs for numbers (e. g., Five Little Ducks), or for farm animals (e.g., Old MacDonald Had a Farm), animals from other semantic subcategories (e.g., jungle or forest), and clothing songs for different semantic subcategories (e.g., “Get Dressed for the Day”). Similar songs are also used in Finland in Finnish. Parents sing these songs or other self-composed songs for their children in everyday life to correspond to an everyday task (e.g., when a child is getting dressed or eating a meal) allowing the child to both build their vocabulary as well as learn the link between an item and the context in which it may be used [71].

As the participants of the present study were at an age where their phonological skills were still developing, the role of the last stages of verbal recall warrants mention. Skills such as finding the phonological codes for the words (phonemic and stress patterns), or finding phonetic-articulatory gestures of the words, were all required for the semantic verbal fluency tasks used in this study [3]. Songs for children tend to be highly repetitive, meaning that when children sing these songs, they typically repeat the same lyrics multiple times. This could help them learn, practice and store the motor programs for target words and their phonological representations, thereby facilitating the verbal recall needed for good performance in verbal fluency tasks [34].

Finally, it is also possible that the children having more informal music had better ability to keep their focused attention on the task. Several studies using attention-related P3a brain responses show links between these responses and informal music activities [58–60]. These studies imply more mature auditory attention and better ability to suppress distracting sounds with more informal music. Thus, the present results on informal music may be partially a consequence of improved attention-related brain networks, improving the ability to focus attention to the semantic verbal fluency task per se, in young children with more informal music activities.

### Limitations and directions for future research

When interpreting the results of this study, a number of methodological considerations should be accounted for. Firstly, this study’s findings are purely correlational, and not causal. That is, although the study found an association between improved verbal semantic fluency and more informal music activities, there could be another factor(s) driving this relationship. It may be that informal music activities helped to improve verbal fluency and language, but it may also be that children with better verbal fluency and language were more inclined to participate in music activities. We also found that the more children participated in formal music activities, the more they were involved with informal music activities. Although the link between formal music activity and verbal fluency did not remain significant in GLM, it is still possible that participation in formal music activities could increase their potential to engage in more informal music activities (e.g., greater interest in music, listening/singing to music stimulated by the formal music training etc.) thereby indirectly contributing to the result found. Although we controlled age and parental education in the GLM, and they did not correlate significantly with the amount of music activities or semantic verbal fluency performance, there may also be other intermediary variables driving the association (e.g., genetic factors, other familial/upbringing considerations such as involvement in other extra-curricular tasks, social network/friendship groups etc., to name just a few possibilities). For instance, there is a genetic contribution to the motivation required to continue with music activities, and to associations between music training and verbal or cognitive ability ([72], for a review). Even singing pitch accuracy has been found to be inﬂuenced by both genes (nature) and the experiences of music and singing in the family and/or in early childhood (nurture) [73]. It is though beyond the scope of this paper to discuss the role of nature vs. nurture in pediatric music development. Future study designs should consider using randomized controlled trials in order to better evaluate whether music activities improve children’s verbal recall and semantic verbal fluency performance with sufficient participants in order to better account for the contribution of key variables such as parental education, age, genes and socioeconomic status on performance. One limitation is that we did not use any standardized developmental scales or measures of language skills to assess the children’s developmental and linguistic status, relying onto on subjective parent report.

It is important to note that parents did the task with their children. The results obtained were comparable to existing studies. For example, Isacoff & Stromswold’s [23] paper adopted a comparable methodology whereby parents were asked to record the number of words listed by their child in an animal verbal fluency task using a 30-second timeframe. In that study children aged 3 years listed an average of 3.51 animals, children aged 4 years named an average of 6.05 animals, and those aged 5 years named an average of 7.61 animals. In the current study using a 60-second time period, children aged 3 years listed on average 6 animals, children aged 4 years listed 9, and children aged 5 years listed 9 animals. To contrast, when the instructions and words listed were recorded by an ‘examiner’ (unfamiliar to the child), such as in Klenberg et al.’s [22] study, the number of listed words were lower. It may be that children feel less stressed or more ‘at ease’ when completing tasks with parents rather than unfamiliar researchers, and the performance in this situation reflects in the best way the ability of the child to retrieve words from the semantic lexicon. Parental-report instruments are increasingly being applied in both clinical and research settings, and the inclusion of caregivers’ observations in clinical decision making has been recommended by the American Academy of Pediatrics (cited in Stolt, [69]). This underlied our methodological decisions on the procedures for measuring verbal fluency performance. The task was quick and easy to conduct for parents and children, and parents could give the instructions to their child to better ensure their child’s understanding of task requirements and also make the assessment less ‘test-like’ and more relaxed.

It is also important to note that the Wilk’s Lambda for the multivariate test was not significant as only two of the independent variables (age and informal music activity) were associated with the dependent variable (semantic verbal fluency performance). This indicates that this cohort of children were not a cohesive group in relation to all of the independent variables in the model, potentially driven by the different distributions of the music variables (substantially greater amount of informal music activity participation than formal). Notably, according to our questions on singing (how often parents sang face to face with their child during the previous year and during the first living year of the child) many parents had sung for their children starting from their first year of life and continued to do this through to when the data was collected. This persistent exposure in very early childhood might have had a cumulative beneﬁt on their child’s neural development at a time when brain plasticity is high [74] (as opposed to music/singing exposure only in the last year, or only when the child was a toddler). Future studies should examine patterns of home music activities from birth, possibly collating a music exposure/participation score accounting for music exposure from birth rather than only in the previous 12 months, although the feasibility of this may be challenging.

## Conclusions

To conclude, the findings of the present study showed that both the quantitative performance (the number of correct words) and qualitative performance (the number of clusters, switches and semantic subcategories) improved with increasing age in children aged 2–5 years, with specific differences between age groups. Interestingly, switches between subcategories had already emerged in some children at the age of 2. The present findings also demonstrated a relationship between better quantitative and qualitative performance (the number of correct words and semantic subcategories) and more informal music activities. These findings make an important contribution to the understudied ﬁeld of the development of young children’s verbal recall in the form of semantic verbal fluency performance, and extend current research findings that not just formal, but additionally informal music participation is associated with quantitative and qualitative semantic verbal fluency performance. Effective verbal recall is essential to everyday communication, the generation of narratives, expressing feelings, answering questions, and writing. The findings of this study should encourage researchers to further investigate the role of informal music activities for improving semantic verbal fluency performance and verbal recall.

## Supporting information

S1 Table The means and standard deviations for each question on music activities and for the sum score “Formal music” per age group and across all children. (PDF)

S2 Table The means and standard deviations for each question on music activities and for the sum score “Informal music” per age group and across all children. (PDF)

S3 Table Spearman correlations between “Parental education” score (sum of maternal and paternal education), age of the child (years, continuous variable), “Formal music” score (sum of formal music activities) and “Informal music” score (sum of informal music activities)‌‌. (PDF)

S4 Table Pairwise comparisons of the number of correct words between different age groups. (PDF)

S5 Table Pairwise comparisons of the number of clusters (N clusters) and the mean cluster sizes (M cluster size) between different age groups. (PDF)

S6 Table Pairwise comparisons of the number of switches between age groups. (PDF)

S7 Table Pairwise comparisons of the number of semantic subcategories between different age groups. (PDF)

S1 File Questions, response options and scales used for statistical analyses addressing hypotheses 1, 2 and 3. (PDF)

S2 File Instructions for parents regarding the semantic verbal fluency tasks. (PDF)

S3 File Data. (XLSX)

S4 File Examples of children’s semantic verbal fluency performances reported by parents. (DOCX)
