# Supplementary material for: Development of semantic verbal fluency in children aged 2 to 5 and its relationship with participating in music activities
Source: PLoS One. 2026 Jun 24;21(6):e0350326. doi: 10.1371/journal.pone.0350326 (PMC13293418; doi:10.1371/journal.pone.0350326)
Supplement: S1 Table — (PDF) [file pone.0350326.s001.pdf]

S1 Table. The means and standard deviations for each question on music activities and for the sum score "Formal music" per age group and across all children.

	Formal music activities variables													
	B7		B8		B9		B10		B11		B12		”Formal music" score	
Age group	*M*	*SD*	*M*	*SD*	*M*	*SD*	*M*	*SD*	*M*	*SD*	*M*	*SD*	*M*	*SD*
2yr olds	0.42	1.26	2.74	2.23	1.53	2.06	1.47	1.98	0.42	1.26	1.21	2.02	7.79	7.03
3yr olds	0.60	1.47	3.30	1.75	1.70	2.08	2.05	2.11	0.80	1.64	0.65	1.50	9.10	5.56
4yr olds	0.43	1.21	3.29	1.68	1.48	1.94	1.33	1.93	1.00	1.73	0.33	1.11	7.86	4.60
5yr olds	1.68	1.95	3.58	1.68	2.11	1.94	1.79	1.96	1.32	1.83	0.89	1.70	11.37	5.05
All	0.77	1.55	3.23	1.83	1.70	1.98	1.66	1.98	0.89	1.63	0.76	1.60	9.00	5.68

8-point Likert scale was utilised (0 = not at all; 1 = less than once in a month; 2 = once in a month; 3 = 2-3 times per month; 4 = weekly; 5 = 2-3 times per week; 6 = 4-6 times per week; 7 = daily. For detailed information on the questions, response options and scales, see S1 File.

B7. Music Lessons;
B8. Singing Groups;
B9. Instrumental Groups;
B10. Special children's music programs;
B11. Dance classes;
B12. Other organized music programs or activities.
"Formal music" score = the sum of responses to the questions B7-B12.
