# Supplementary material for: Development of semantic verbal fluency in children aged 2 to 5 and its relationship with participating in music activities
Source: PLoS One. 2026 Jun 24;21(6):e0350326. doi: 10.1371/journal.pone.0350326 (PMC13293418; doi:10.1371/journal.pone.0350326)
Supplement: S2 Table — (PDF) [file pone.0350326.s002.pdf]

S2 Table. The means and standard deviations for each question on music activities and for the sum score “Informal music” per age group and across all children.

	Informal music activities variables													
	B15		B16		B17		B18		B19		B20		B21	
Age group	*M*	*SD*	*M*	*SD*	*M*	*SD*	*M*	*SD*	*M*	*SD*	*M*	*SD*	*M*	*SD*
2yr olds	6.63	0.68	3.74	2.84	4.47	1.61	3.58	1.74	0.26	1.15	2.61	2.03	5.58	2.24
3yr olds	6.50	0.83	3.10	2.57	5.20	1.47	2.20	2.17	1.15	1.98	3.25	2.10	5.75	1.48
4yr olds	6.52	0.75	4.14	2.33	4.48	1.81	3.38	2.48	0.62	1.12	2.86	1.71	5.43	1.47
5yr olds	6.74	0.56	3.68	2.33	5.42	0.90	2.74	2.23	2.16	2.09	3.41	2.24	5.63	1.80
All	6.59	0.71	3.67	2.50	4.89	1.53	2.97	2.21	1.04	1.76	3.03	2.00	5.59	1.74

	B22		B23		B5		B6		H1		”Informal music” score	
Age group	*M*	*SD*	*M*	*SD*	*M*	*SD*	*M*	*SD*	*M*	*SD*	*M*	*SD*
2yr olds	5.47	1.17	0.42	0.51	6.74	0.56	6.63	0.60	6.37	1.67	52.94	4.73
3yr olds	4.50	1.88	0.80	0.52	6.15	1.50	6.40	0.94	6.40	1.43	51.40	8.27
4yr olds	4.76	1.76	0.67	0.48	6.48	0.81	6.90	0.44	6.19	1.47	52.43	9.47
5yr olds	4.74	1.69	1.00	0.33	5.68	1.67	6.47	0.90	6.47	0.90	54.41	7.51
All	4.86	1.66	0.72	0.50	6.27	1.26	6.61	0.76	6.35	1.38	52.72	7.71

8-point Likert scale was utilised (0 = not at all; 1 = less than once in a month; 2 = once in a month; 3. = 2-3 times per month; 4 = weekly; 5 = 2-3 times per week; 6 = 4-6 times per week; 7 = daily. For detailed information on the questions, response options and scales, see S1 File.
B15. Listening to music informally (audio only);
B16. Social music activities;
B17. Musical videos;
B18. Family music activities;
B19 Music online (games, listening. etc.);
B20. Independent music exploration;
B21. Creating/making up songs or music performances for play or fun;
B22. Dancing informally;
B23. Live music concerts;
B5. Parental singing face to face with their child during the previous year;
H1. Singing by the child in general;
“Informal music” score = the sum of the responses to the questions B15-B23 and B5, B6 and H1.
