# Supplementary material for: Development of semantic verbal fluency in children aged 2 to 5 and its relationship with participating in music activities
Source: PLoS One. 2026 Jun 24;21(6):e0350326. doi: 10.1371/journal.pone.0350326 (PMC13293418; doi:10.1371/journal.pone.0350326)
Supplement: S4 Table — (PDF) [file pone.0350326.s004.pdf]

S4 Table. Pairwise comparisons of the number of correct words between different age groups.

Age group	Animals *z*	Animals p^b	Clothes *z*	Clothes p^b
2- vs. 3yr olds	-2.756	.035*	2.165	.182
2- vs. 4yr olds	-5.003	<.001***	4.989	<.001***
2- vs. 5yr olds	-5.896	<.001***	5.031	<.001***
3- vs. 4yr olds	-2.244	.149	2.906	.022*
3- vs. 5yr olds	-3.215	.008*	2.976	.018*
4- vs. 5yr olds	-1.038	.299	-.913	1.000

Pairwise comparisons have been calculated using 1) Kruskal-Wallis test 2) post hoc -tests with Dunn test; z = standardized difference of the mean of ordinal numbers; * = $p < .05$; ** = $p < .01$; *** = $p < .001$; p^b = Bonferroni-corrected *p*.
