# Supplementary material for: Development of semantic verbal fluency in children aged 2 to 5 and its relationship with participating in music activities
Source: PLoS One. 2026 Jun 24;21(6):e0350326. doi: 10.1371/journal.pone.0350326 (PMC13293418; doi:10.1371/journal.pone.0350326)
Supplement: S5 Table — (PDF) [file pone.0350326.s005.pdf]

S5 Table. Pairwise comparisons of the number of clusters (N clusters) and the mean cluster sizes (M cluster size) between different age groups.

Age group	Animals z	Animals p^b	Clothes z	Clothes p^b
N clusters				
2- vs. 3yr olds	-1.485	.825	-2.341	.115
2- vs. 4yr olds	-3.996	<.001***	-4.783	<.001***
2- vs. 5yr olds	-4.890	<.001***	-5.274	<.001***
3- vs. 4yr olds	-2.527	.069	-2.542	.066
3- vs. 5yr olds	-3.467	.003**	-3.019	.015*
4- vs. 5yr olds	-1.015	1.000	.435	1.000
M cluster size				
2- vs. 3yr olds	-.439	1.000	-.522	1.000
2- vs. 4yr olds	-1.555	.720	-1.751	.480
2- vs. 5yr olds	-2.407	.096	-1.754	.476
3- vs. 4yr olds	-1.287	1.000	-1.642	.603
3- vs. 5yr olds	-2.270	.139	-1.643	.603
4- vs. 5yr olds	-1.027	1.000	-.024	1.000

Pairwise comparisons have been calculated using 1) Kruskal-Wallis test 2) post hoc -tests with Dunn test; z = standardized difference of the mean of ordinal numbers; * = $p < .05$; ** = $p < .01$; *** = $p < .001$; p^b = Bonferroni-corrected *p*.
