# Supplementary material for: Development of semantic verbal fluency in children aged 2 to 5 and its relationship with participating in music activities
Source: PLoS One. 2026 Jun 24;21(6):e0350326. doi: 10.1371/journal.pone.0350326 (PMC13293418; doi:10.1371/journal.pone.0350326)
Supplement: S6 Table — (PDF) [file pone.0350326.s006.pdf]

S6 Table. Pairwise comparisons of the number of switches between age groups.

Age group	Animals z	Animals p^b	Clothes z	Clothes p^b
2- vs. 3yr olds	-2.772	.033*	-3.043	.014*
2- vs. 4yr olds	-3.991	.001***	-4.289	.001***
2- vs. 5yr olds	-4.114	.001***	-4.667	.001***
3- vs. 4yr olds	-1.270	1.000	-1.321	.569
3- vs. 5yr olds	-1.327	1.000	-1.670	1.000
4- vs. 5yr olds	.025	1.000	.327	1.000

Pairwise comparisons have been calculated using 1) Kruskal-Wallis test 2) post hoc -tests with Dunn test; z = standardized difference of the mean of ordinal numbers; * = $p < .05$; ** = $p < .01$; *** = $p < .001$; p^b = Bonferroni-corrected p.
