# Supplementary material for: Development of semantic verbal fluency in children aged 2 to 5 and its relationship with participating in music activities
Source: PLoS One. 2026 Jun 24;21(6):e0350326. doi: 10.1371/journal.pone.0350326 (PMC13293418; doi:10.1371/journal.pone.0350326)
Supplement: S7 Table — (PDF) [file pone.0350326.s007.pdf]

S7 Table. Pairwise comparisons of the number of semantic subcategories between different age groups.

Age group	Animals z	Animals p^b	Clothes z	Clothes p^b
2- vs. 3yr olds	-1.404	.962	-2.303	.128
2- vs. 4yr olds	-3.191	.009**	-4.910	<.001***
2- vs. 5yr olds	-4.852	<.001***	-5.308	<.001***
3- vs. 4yr olds	-1.794	.437	-2.710	.012*
3- vs. 5yr olds	-3.510	.003**	-3.092	.040*
4- vs. 5yr olds	-1.781	.449	.339	1.000

Pairwise comparisons have been calculated using 1) Kruskal-Wallis test 2) post hoc -tests with Dunn test; z = standardized difference of the mean of ordinal numbers; * = $p < .05$; ** = $p < .01$; *** = $p < .001$; p^b = Bonferroni-corrected p.
