# Supplementary material for: Development of semantic verbal fluency in children aged 2 to 5 and its relationship with participating in music activities
Source: PLoS One. 2026 Jun 24;21(6):e0350326. doi: 10.1371/journal.pone.0350326 (PMC13293418; doi:10.1371/journal.pone.0350326)
Supplement: S1 File — (PDF) [file pone.0350326.s008.pdf]

S 1. Questions, response options and scales used for statistical analyses addressing hypotheses 1, 2 and 3.

To help the reader, the questions and response options have been translated from Finnish to English language for this Appendix. Please note that the wording below are not verbatim translations, but instead translations that give the reader the closest approximation (in grammatically correct English) to the Finnish question.

Socioeconomic background

A3. Family income per year before tax.
Response options for parents: 1 = 20 000 € or less, 2 = 20 001 - 35 000 €, 3 = 35 001 - 50 000 €, 4 = 50 001 - 65 000 €, 5 = 65 001 € or more, (Likert scale 1-5).

A4. Education of mother - if the family has two mothers/female caretakers, please choose the level of education for the mother with higher education.
Response options for parents: 1 = primary school, 2 = middle school, 3 = lower university, 4 = middle university, 5 = upper university, 6 = graduate university (PhD) degree, (Likert scale 1-6).

A5. Education of father - if the family has two fathers/male caretakers, please choose the level of education for the father etc. with higher education.
Response options for parents: 1 = primary school, 2 = middle school, 3 = lower university, 4 = middle university, 5 = upper university, 6 = graduate university (PhD) degree, (Likert scale 1-6).

Age

A16. Current Age of the child: (X) years and (Y) months.
The questions above were used to calculate the chronological age of the participants.

Questions on music activities

Please note that for all music activity questions, the logic of the survey was programmed so that the second question on the frequency of participation was only asked if the parent answered ‘yes’ to the first question as to whether the child participated in that particular activity (yes/no).

Response options for the second question were: 1 = less than once in a month; 2 = once in a month; 3 = 2-3 times per month; 4 = once in a week; 5 =2-3 times per week; 6 = 4-6 times per week; 7= daily.

If the parent answered no to the first question (please see below), then a score of ‘0’ was allocated for statistical analyses for this child, leading to an 8-point Likert scale 0-7.

Informal music activities

B15 Listening to music informally (audio only):

B15.1 Has your child ever listened to music at home or elsewhere from recordings (without visual support, e.g. in the car, when going to bed, while eating, while playing, during naps, etc.)?

B15.2 How often has your child listened to music?

B16. Social music activities:

B16.1 Has your child ever participated in social (self-initiated, not directed) musical activities (e.g. imitation, making music in role-plays with friends)?

16.2 How often has your child participated in social music activities?

B17. Musical videos:

B17.1 Has your child ever watched music programs or music videos (e.g. music episodes of children's programs, YouTube videos, etc.)?

B17.2 How often has your child watched music programs or videos?

B18. Family music activities:

B18.1 Has your family ever made music together (for example, sing or play)?

B18.2 Has your child participated in making music together?

B18.3 How often have you made music together?

B19 Music online (games, listening, etc.):

B19.1 Has your child ever used online music programs or games?

B19.2 How often has your child used the aforementioned programs or games?

B20. Independent music exploration:

B20.1 Has your child ever explored music independently (e.g. played self-made instruments or bowls/pots, etc.)?
B20.2 How often has your child made independent musical explorations?

B21. Creating/making up songs or music performances for play or fun:
B21.1 Has your child ever sung songs (learned or self-invented) or made musical performances during playtime?
B21.2 How often has your child sung or made musical performances during playtime?

B22. Dancing informally:
B22.1 Has your child ever danced to the music on his own initiative?
B22.3 How often your child has danced to the music on his own initiative?

B23. Live music concerts:
B23.1 Has your child ever been to music concerts (for example, in connection with children's events, concerts of children's bands, music festivals)?
B23.2 How often your child has been in these concerts?

Questions on singing:
B5. How often have the child's parents sung face to face with their child during the previous year?
B6. How often have the child's parents sung face to face with their child during the child's first year of life?
H1. How often does the child sing at home in general (alone, with siblings or friends or parents etc., altogether)?

Formal music activities

B7. Music Lessons:
B7.1 Has your child ever taken music instrument or singing lessons (such as individual or group lessons, musical instrument training)?
B7.2 How often has your child have these instrument or singing lessons?

B8. Singing Groups:
B8.1 Has your child ever sung in a formal music group (e.g. music play school, parish club, choir)?

B8.2 How often has your child sung in a group?

B9. Instrumental groups:

B9.1 Has your child ever played in a form music instrument group (e.g., band, music group, orchestra, etc.)?

B9.2 How often has your child played in a music instrument group?

B10. Special children's music programs:

B10.1 Has your child ever participated in music or music education programs aimed at children (e.g. in municipal or private music schools, their music instrument groups or instrument training, Jamkids, the Suzuki method, the Kodaly method, "mini-maestros")?

B10.2 How often has your child participated in these?

B11. Dancing classes:

B 11.1. Has your child ever participated in dancing classes?

B12.2. How often has your child participated in dancing lessons?

B12. Other organized music programs or activities:

B 12.1 Has your child ever participated in other musical activities organized by preschool, church, etc.?

B12.2. How often has your child participated in these activities?
