# Supplementary material for: Development of semantic verbal fluency in children aged 2 to 5 and its relationship with participating in music activities
Source: PLoS One. 2026 Jun 24;21(6):e0350326. doi: 10.1371/journal.pone.0350326 (PMC13293418; doi:10.1371/journal.pone.0350326)
Supplement: S2 File — (PDF) [file pone.0350326.s009.pdf]

S2 File. Instructions for parents regarding the semantic verbal fluency tasks.

Please note that this is a conversational English translation from the Finnish instructions– i.e. it is not a verbatim translation word-for-word of the Finnish instructions

The following instruction was given in the questionnaire for parents for the two semantic categories used in this study, animals and clothes (instruction given after that for animals):

"Ask the child to list the animals/clothes for exactly one minute. The purpose is for the child to list the animals/clothes from memory. It is important that you don't show the child pictures of animals/clothes, toys, or gestures, or whisper or name animals/clothes, etc. Also, don’t help the child, e.g., by asking to repeat animals after you, or by describing them. So, guide your child only with the following task instructions: *“Tell me all the animals/clothes there are. They can be any kind of animals/clothes, but don’t say the same animal/clothe twice. And try to come up with as many animals/clothes as possible. What are all the animals/clothes you know?”*

Measure the time, e.g., with a mobile phone stopwatch or timer, etc. Record the child's production (on a mobile phone, computer, etc.). If the child stops listing, at 30 seconds you can tell him/her "what other animals/clothes do you know?" If the child keeps quiet or waits for confirmation of his/her actions, you can give him/her feedback "hmm", "yeah". From the recording, please write into the text box all of the speech produced by the child during that one minute -both ‘sounds’ (such as uh, woof woof,) as well as actual words (e.g. dog, cat, flower, pants, shirt, sock, flower). Also include all of the words the child’s repeats (e.g. "doggie, doggie"/ "pants, pants"), or sentences said by the child (e.g. "I know more".). If the child doesn't say a word or doesn't understand the task instruction, indicate this in the place which asks for an answer.”
