# Supplementary material for: Development of semantic verbal fluency in children aged 2 to 5 and its relationship with participating in music activities
Source: PLoS One. 2026 Jun 24;21(6):e0350326. doi: 10.1371/journal.pone.0350326 (PMC13293418; doi:10.1371/journal.pone.0350326)
Supplement: S4 File — (DOCX) [file pone.0350326.s011.docx]

**S4 File. Examples of children’s semantic verbal fluency performances reported by parents.**

An example of children aged between 2 years 0 months - 2 years 5 months:

Animals: ”Ankka, oaoea, lehmä, toinen ankka, kaksi ankkaa, emo ja poikasia”

In English: “Duck, oaoea, cow, another duck, two ducks, mother and chicks.”

Clothes: ”Pipo, haalari, villapuku, fleecetakki, kauluri ja toinen villapuku. Oli näitä.”

In English: ”Beanie, overall, wool suit, fleece jacket, neck warmer, and another wool suit. There were these.”

An example of children aged between 2 years 6 months - 2 years 11 months:

Animals: “Hauhau, kissa, lintu”

In English: “Woof woof, cat, bird”

Clothes: “Pipo hattu”

In English: “Beanie hat”

An example of children aged between 3 years 0 months – 3 years 5 months:

Animals: ”Mitä? öö, mm, krokotiileja, mm, lehmiä, öö, virtahepoja, hevosia, öö öööö, possuja, ööö sikoja, ööö ääää, rekkuja, ääää, isiä”

In English: “What? um, mm, crocodiles, mm, cows, um, hippos, horses, um um um, pigs, um um sows, um um um, doggies, um, dads”

Clothes: ”Öööö, muumimekko, äääää mmm, prinssimekko. äää sukkia. sukkia, mmm vaatteita, isejä, ööö rikuja, prinssejä, mmmm tyynyjä“

In English: “Um um um, Moomin dress, um, princess dress, um socks, socks, mm clothes, dads, um, rikus, princes, mmm pillows”

An example of children aged between 3 years 6 months – 3 years 11 months:

Animals: ”Hmmm kissa, tiikeri, äititiikeri, karhu ja jaaa lintuja, hiiri, jaaa tuota heppa ja jäniksiä jaa mitä sanoisin.. mesikämmen, itse asiassa kala, hauki, ahven. Ja vielä yksi: merenneito. (Viimeinen lause tuli kun olin pyytänyt lopettamaan)”

In English: “Hmm cat, tiger, mother tiger, bear and and birds, mouse, and um horse and rabbits and um what should I say... honey bear, actually fish, pike, perch. And one more: mermaid.” (The last sentence came when I had asked to stop)”

Clothes: ”Takki, paita, ja henkselit, henks -se -lit, jaa mekko, teeppari, ymmm.. mä kuiskaan sulle: housut jaa… (Teeppari = t-paita, käyttämämme nimitys)”

in English: “Jacket, shirt, and suspenders, susp -en -ders, jaa dress, t-shirt, um... I’ll whisper to you: pants and um... (Teeppari = t-shirt, the term we use)”

An example of children aged between 4 years 0 months – 4 years 5 months:

Animals: “Aasi, hevonen, jääpingiini, lehmä, hylje, norppa, lepakko, sinitiainen, ja ja ja tota, tota tota härkä ja peura, tota ehkä mä osaan vielä yhen, hirvi, tota kotka ja pöllö”

In English: “Donkey, horse, ice penguin, cow, seal, saimaa ringed seal, bat, blue tit, and and um bull and deer, um maybe I can name one more, moose, um eagle and owl”

Clothes: ”Huppari, housut, sukat, paita, pipo, takki ja pusero ja kaulahuivi, pipo, mutta en mä tiedä mitä muita vaatteita on olemassa, kauluri ja kypärämyssy, niin monta, ehkä mä osaan sanoo vielä pari, pusero ja villahousut”

In English: “Hoodie, pants, socks, shirt, beanie, jacket, and blouse and scarf, beanie, but I don’t know what other clothes exist, neck warmer and helmet hat, so many, maybe I can name a couple more, blouse and wool pants”

An example of children aged between 4 years 0 months – 4 years 5 months:

Animals: ”Mmm leijona. Muurahaisia, ööö tiikereitä, ääämm lentomuurahaisia. Jaaaaa joutsenia ja leppäkerttuja. Mmmm kirahveja mmm äää seeproja. Mmm ää En muista muita. *aika täyttyi samalla”

In English: “Mmm lion. Ants, um tiger, um flying ants. And swans and ladybugs. Mmm giraffes, mmm um zebras. Mmm um I can’t remember any others.” *time ran out at the same time*”*

Clothes: “Ääää haalarin äää rukkaset, lapaset äääm ööömömömmmm mitä muita on olemassa… no rupesin nyt miettimään. No villasukka, pipo... yymmmm äää ääämmm. Paita...sukkahousut”

In English: “Um overall, um mittens, gloves, um um um what else exists... well, I started thinking now. Well wool socks, beanie... um um um. Shirt... tights”

An example of children aged between 5 years 0 months – 5 years 5 months:

Animals: ”Ööö... kissa, koira, puppuppupu, sitten leijona, lintu, jaaaa koira, lehmä ja possu ja hevonen”

In English: “Um... cat, dog, puppuppupu, then lion, bird, and um dog, cow, and pig and horse”

Clothes: ”Hmmm paita, housu, sukat, Äh mekko, jaaa pipo ja hanska ja haalari”

In English: “Hmm shirt, pants, socks, um dress, and um beanie and glove and overall”

An example of children aged 5 years 6 months – 5 years 11 months:

Animals: ”Kiraffi, gorilla, apina jaa öö karhu, pandakarhu, jääkarhu jaa haisunäätä, näätä jaa myyrä, hiiri ee delfiini, valas, kala, ahven, jaa yy, poro, hirvi.”

In English: “Giraffe, gorilla, monkey and um bear, panda bear, polar bear and um skunk, marten and um mole, mouse ee dolphin, whale, fish, perch, and um, reindeer, moose.”

Clothes: “Kalsarit ja tota sitten paita, housut ja öö sitten vielä haalari, ulkohaalari ja sitten öö ulkohousut ja takki lopeta jaa pipo ja hanskoja ja mmmmm.”

In English: ”Underwear and um then shirt, pants and um then also overall, outdoor overall and then um outdoor pants and jacket stop and um beanie and gloves and mmmmm.”
